# Prevalence of substance use and knowledge of its effects among secondary school students in Lagos, Nigeria

**DOI:** 10.4102/sajpsychiatry.v31i0.2370

**Published:** 2025-05-30

**Authors:** Tolulope O. Kolawole, Adedoyin O. Ogunyemi, Alexander R. Lucas

**Affiliations:** 1 Department of Social and Behavioural Sciences, School of Public Health, Virginia Commonwealth University, Virginia, United States of America; 2 Department of Community Health and Primary Care, College of Medicine, University of Lagos, Lagos, Nigeria

**Keywords:** substance use, drugs, adolescents, youths, students, psychoactive substances, alcohol, marijuana, tramadol

## Abstract

**Background:**

World Health Organization defines substance use as harmful or hazardous use of psychoactive substances, including alcohol and illicit drugs. There is an increased rate of substance use among youths and adolescents. Substance use significantly increases the risk for mental disorders.

**Aim:**

The aim of this study was to determine the prevalence of substance use and knowledge of its effects among secondary school students in Lagos, Nigeria.

**Setting:**

The study was conducted among Secondary schools in Lagos, Nigeria.

**Methods:**

We conducted a cross-sectional, descriptive study among 800 participants who were selected using a multi-stage sampling method. The instrument for data collection was the amended WHO students’ drug-use questionnaire. The data were analysed using Epi Info 7 software.

**Results:**

The lifetime prevalence of substance use was 13.6%, while current users’ prevalence was 6.9%. Apart from alcohol consumption, the substance most frequently used was tramadol (52.7%), followed by marijuana (36.4%). Almost all the participants (98.1%) were aware of substance use. Most students (88.7%) were able to identify the effects of substance use, including short-term complications (79.1%) and long-term complications (61.1%).

**Conclusion:**

The high prevalence of alcohol and tramadol use among the participants underscores the need for stricter government policies on the accessibility of these products to adolescents and the need for increased awareness of their effects.

**Contribution:**

The lower knowledge of long-term complications of substance use (61.1%) in comparison to short-term complications (79.1%) underscores the need for further research and increased advocacy on long-term complications of substance use among youths and adolescents.

## Introduction

### Background

The World Health Organization (WHO) defines substance use as the ‘harmful or hazardous use of psychoactive substances, including alcohol and illicit drugs’.^1^ These substances can lead to dependence syndrome, characterised by a range of behavioural, cognitive and physiological effects that develop with repeated use.^2^ This includes a strong urge to use the substance, difficulty in controlling its use despite negative consequences, prioritising substance use over other activities, increased tolerance and sometimes experiencing physical withdrawal symptoms.^2^ Among adolescents and young adults in secondary schools, the use of alcohol, tobacco and other substances is a significant risk-taking behaviour.^2^ Despite global awareness and education efforts about psychoactive substances, many adolescents remain unaware of the potential adverse consequences. Factors such as curiosity, social pressure and influence from peer groups are commonly cited reasons for engaging in substance use.^2^

According to the United Nations Office on Drugs and Crime (UNODC) World Drug Report 2023, drug use remains prevalent globally.^3^ In 2021, approximately 1 out of every 17 individuals aged 15–64 worldwide reported using drugs within the past year.^3^ The estimated number of users has risen from 240 million in 2011 to 296 million in 2021, constituting 5.8% of the global population in that age group. This represents a 23% increase, largely influenced by population growth.^3^ Cannabis retains its status as the most used drug, with an estimated 219 million users globally in 2021, accounting for 4.3% of the adult population worldwide. Usage trends show an upward trajectory and while men still comprise the majority of cannabis users globally (about 70%), the gender gap is narrowing in certain regions; women now represent 42% of cannabis users in North America.^3^

According to the report, in 2021, it was estimated that 36 million people had used amphetamines, 22 million had used cocaine, and 20 million had used substances similar to ‘ecstasy’ within the previous year. Female participation is notably higher among users of amphetamine-type stimulants (45%) and non-medical pharmaceuticals (between 45% and 49%), whereas the majority of users of opiates (75%) and cocaine (73%) are male.^3^ Opioids continue to pose the greatest risk in terms of severe drug-related harm, including fatal overdoses. The report highlights that an estimated 60 million people engaged in non-medical opioid use in 2021, with 31.5 million primarily using opiates such as heroin.^3^

In Nigeria, the legal age for purchasing and consuming alcohol is 18 years,^4^ although this regulation is hardly enforced.^5^ Tramadol, a commonly abused substance, is classified as a controlled drug in Nigeria as listed in the National Drug Law Enforcement Agency (NDLEA) Act.^6^ Marijuana is an illegal substance, and its use, possession and trafficking are punishable under Nigerian law,^7^ although there have been discussions about its potential decriminalisation.

The United Nations defines adolescence as the phase of life between childhood and adulthood, from ages 10–19^8^ and ‘youths’, as those persons between the ages of 15 and 24 years.^9^ There has been an alarming increase in substance use among youths and adolescents, which poses a substantial risk for developing substance use disorders and potentially other mental illnesses later in life.^10^ Research indicates a high prevalence of comorbidity between substance use disorders and mental health conditions among adolescents.^10^ For instance, a significant proportion of adolescents receiving treatment for substance use disorders also met criteria for other mental illnesses.^11^ Substance use typically begins during adolescence, a critical period when the brain is still developing.^12^ This developmental stage increases vulnerability to substance use and the development of substance use disorders, particularly impacting executive functions such as decision-making and impulse control.^12^

Studies conducted in various African countries, including Nigeria, indicate a rising incidence and decreasing age of onset of substance use.^13,14,15^ This underscores the urgent need for ongoing research to address this issue comprehensively within society.^13,14,15^ Furthermore, while numerous studies have explored drug and substance abuse among Nigerian youths and adolescents^13,14,15^ there remains a gap in understanding the knowledge of the effects of substance use among secondary school students. This study aims to assess both the prevalence of substance use and knowledge of the effects of substance use among secondary school students in Lagos State, Nigeria. The findings will inform future research directions, policy development and intervention strategies.

## Research methods and design

### Study design

This study was a cross-sectional study aimed at characterising the prevalence of substance use and knowledge of its effects, specifically among secondary school students in Lagos State, Nigeria.

### Setting

The study was conducted among secondary schools in Education District 1, Lagos State, Nigeria. Education District 1 in Lagos State comprises both urban and peri-urban schools, reflecting the diverse urbanisation levels of the region. Specific classifications of all schools within the district are not readily available. Regarding student demographics, data from the Lagos State School Census Report for the 2018–2019 academic year indicate that across all educational levels in Lagos State, female students slightly outnumber male students.^16^ Lagos is a major financial centre in Africa, and it is the economic hub of Nigeria. The Mega City has the fourth-highest gross domestic product (GDP) in Africa^17,18^ and houses one of the largest and busiest seaports on the continent.^19,20,21^ It is one of the fastest growing cities in the world.^22^

### Study population and sampling strategy

The study was conducted between March and September 2022. The study population was secondary school students in Lagos State, Nigeria. The study included secondary school students in public senior secondary schools in Lagos State who consented to be a part of the study. The study excluded students who were unable to make informed decisions because of severe health, functional or cognitive impairment. The sample size for the study was determined through the Cochran formula for prevalence studies^23^ using the highest life-time drug use prevalence rate of 40.3% among secondary school students in a recent study^24^ Accounting for attrition loss and the extremely large study population, the sample size of 820 participants was used for this study. A multi-stage sampling method was used for the study. Lagos State has six education districts. Using a simple random sampling method, Education District 1, which covers Agege, Ifako-Ijaiye and Alimosho Local Government Areas, was selected for the study.^25^ There is a total of 41 (forty-one) senior secondary schools in Education District 1.^25^ Twenty percent of these schools, approximately eight schools, were selected for the study through a simple random sampling method. With a total sample size of 820 and a total number of eight participating schools, the number of participants per school was determined by dividing the total sample size by the number of participating schools (820/8 = 102.5). Thus, 102 students were selected in each of the first four sets of schools, while 103 students were selected in each of the remaining four schools. The selection of actual participants in each school was determined by a systematic random sampling method. Using the systematic random sampling method, every 10th student was selected in each school for the study until the sample size allocated to the particular school was attained.

### Data collection techniques

Data were collected through an adapted and well-expanded WHO students’ drug-use questionnaire, which has been previously validated in Nigeria.^26^ The questionnaire and the raw data generated are available on request. The questionnaire was available in English language only. The study utilised paper questionnaires that were self-administered. Each student completed the questionnaires privately within the school environment and returned them to the research assistants once completed. On average, it took each student about 45 min to complete the questionnaire. The questionnaire is divided into three primary sections. Section A, titled ‘Demographic Profile’, is designed to collect essential demographic information about the participants, including their age, gender, religion and educational background. Section B of the questionnaire focused on general knowledge about substance use and the effects of substance use among students. Section C of the questionnaire focused on the knowledge and prevalence of some specific drugs commonly abused among students. The questionnaires were administered by six trained research assistants. The trained research assistants worked with the school administrators of each school to recruit participants for the study using the multi-stage sampling method as described earlier.

### Data analysis

Data collected were analysed using Epi Info software (version 7). Descriptive statistics of frequency, percentage, mean and standard deviation were used to summarise the results. Categorical variables such as gender and class were presented as frequency tables and figures such as bar chart, pie chart etc. The chi-squared test was used to determine the association between variables. Level of significance was set at *p* < 0.05.

### Ethical considerations

Ethical clearance to conduct this study was obtained from the Lagos University Teaching Hospital Health Research Ethics Committee (No. ADM/DSCST/HREC/ APP/4309). Official approval was sought and received from the management of Lagos State Education District 1. The details of the study were fully explained to all the students and only students who consented to be a part of the study were included in the study. A written informed consent was obtained from participants who are above 18 years of age. A written parental consent and a written participant assent were obtained for participants under 18 years of age. The privacy of the participants and confidentiality of the information obtained were strictly ensured. There are no immediate or long-term risks to the participants because of this study.

## Results

In this study, 800 senior secondary school students completed their questionnaires out of the 820 questionnaires administered, resulting in a response rate of 97.6%. The mean age of the participants was 15.3 ± 1.5 years with a range of 12–27 years. Over half of the participants were male (52.5%). All were Nigerian (100.0%). The majority of the participants (70.0%) were Christians (Table 1). The mean family size of the participants was 5.5 ± 1.7 years, with a range of 3–24. Most of the participants (81.8%) had a family size within the range of 3–6. Over a third of the participants (37.0%) had secondary education as the fathers’ highest level of education. Over two-fifths of the participants (43.0%) had secondary education as the mothers’ highest level of education (Table 2).

**TABLE 1:** Socio-demographic characteristics (*N* = 800).

Variable	Frequency	Range	Mean	s.d.	%
**School**					
School 1	97	-	-	-	12.2
School 2	101	-	-	-	12.6
School 3	105	-	-	-	13.1
School 4	105	-	-	-	13.1
School 5	104	-	-	-	13.0
School 6	85	-	-	-	10.6
School 7	108	-	-	-	13.5
School 8	95	-	-	-	11.9
**Age (years)**					
12–14	259	-	-	-	32.4
15–17	484	-	-	-	60.5
≥ 18	57	-	-	-	7.1
**Range**	-	12–27	-	-	-
Mean ± s.d.	-	-	15.3	1.5	-
**Class**					
SS1	400	-	-	-	50.0
SS2	261	-	-	-	32.6
SS3	139	-	-	-	17.4
**Sex**					
Male	420	-	-	-	52.5
Female	380	-	-	-	47.5
**Religion**					
Christianity	560	-	-	-	70.0
Islam	228	-	-	-	28.5
Traditional	12	-	-	-	1.5

s.d., standard deviation; SS, Senior Secondary.

**TABLE 2:** Family size and parents’ educational level of participants (*N* = 800).

Variable	Frequency	Range	Mean	s.d.	%
**Family size**					
3–6	654	-	-	-	81.8
7–9	129	-	-	-	16.1
≥ 10	17	-	-	-	2.1
**Range**	-	3–24	-	-	-
Mean ± s.d.	-	-	5.5	1.7	-
**Father’s education level**					
No formal education	35	-	-	-	4.4
Primary	69	-	-	-	8.6
Secondary	296	-	-	-	37.0
Tertiary	224	-	-	-	28.0
I don’t know or not sure	176	-	-	-	22.0
**Mother’s education level**					
No formal education	36	-	-	-	4.5
Primary	20	-	-	-	2.5
Secondary	344	-	-	-	43.0
Tertiary	208	-	-	-	26.0
I don’t know or not sure	192	-	-	-	24.0

s.d., standard deviation.

**FIGURE 1:**
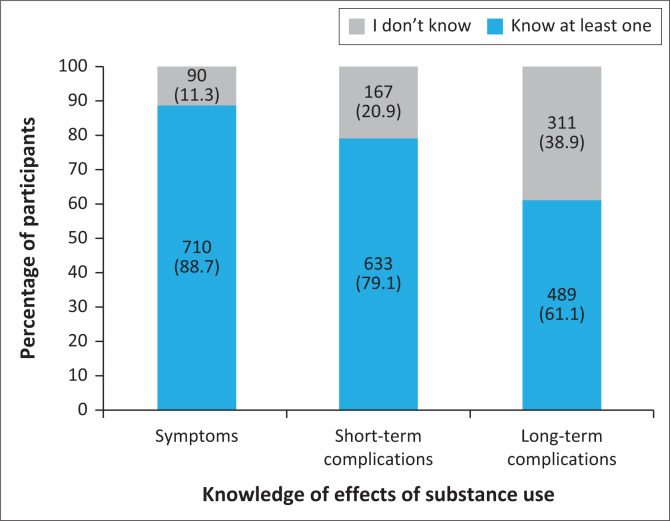
Knowledge of the effects of substance use among participants (*n* = 800).

**FIGURE 2:**
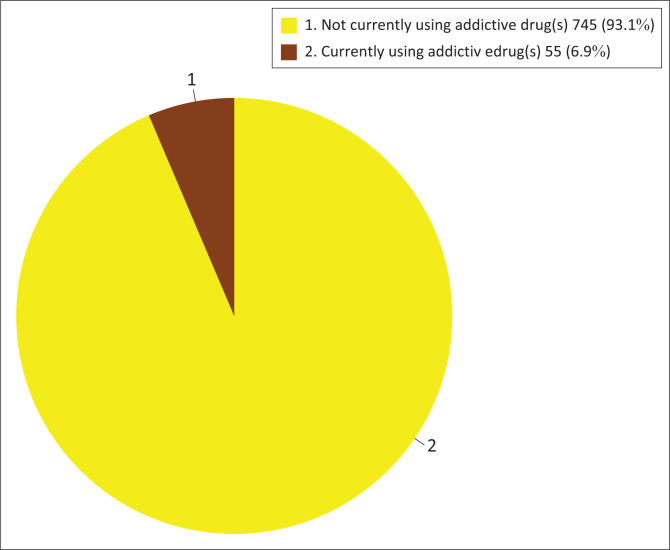
Prevalence of addictive substance use (current users only) (*n* = 800).

**FIGURE 3:**
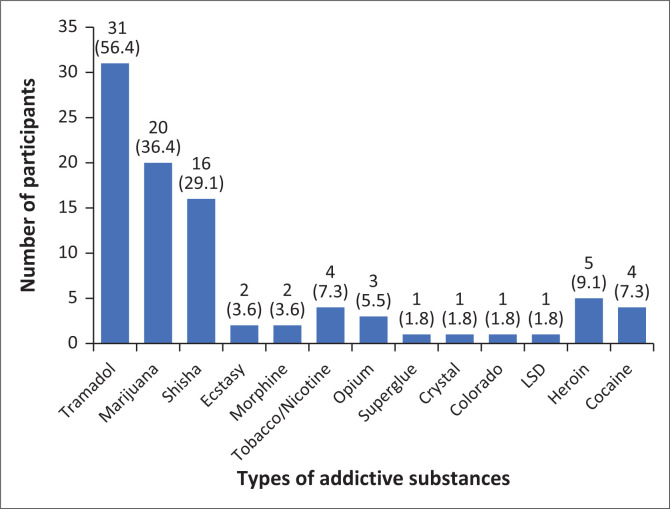
Addictive drugs currently being used by participants (*n* = 55). (Multiple responses allowed).

The lifetime prevalence of substance use among the participants was 13.9% (Figure 1). The current prevalence of substance use among the participants was 6.9% (Figure 2). Substances currently being used by the participants included tramadol (52.7%), marijuana (36.4%), shisha (29.1%), heroin (9.1%), cocaine (7.3%), tobacco or nicotine (7.3%), ecstasy (3.6%), morphine (3.6%), opium (5.5%), superglue (1.8%), crystal meth (1.8%), colorado (1.8%) and LSD (1.8%) (Figure 3).

Over a fifth (22.6%) of all the participants had drunk more than a sip of alcohol, with almost half (51.9%) having drunk alcohol within the past 12 months. The highest frequency (44.2%) of participants who drank alcohol started within the age range of 13–14 years (Table 2).

Almost all the participants (98.1%) had received information on substance use. Effects of substance use most frequently highlighted by the participants included kidney failure (67.5%), brain damage (65.0%), dry mouth (35.9%), visual hallucinations (21.1%) and auditory hallucinations (15.6%). Over a tenth of the participants (11.3%) did not report being aware of the negative effects of substance use. Short-term complications of substance use reported by the participants included dependence (30.8%), anxiety and depression (23.5%), sleep disorder (21.9%), aggressiveness (16.4%), personality disorder (12.4%) and forgetfulness (12.3%). Just over a fifth of the participants (20.9%) did not report knowing of any short-term complications of drug use. Long-term complications of substance use were similarly identified and included dependence (35.0%), anxiety and depression (16.8%), personality disorder (15.6%), aggressiveness (13.8%) and sleep disorder (10.8%). Over a third of the participants (38.9%) did not report knowing of any long-term complications associated with substance use. The majority of the participants were able to identify effects (88.7%), short- (79.1%) and long-term complications (61.1%) of substance use (Figure 1 and Table 3).

**TABLE 3:** Knowledge of the effects of substance use among participants (*N* = 800).

Variable	Frequency	%
**Received information on drug use**		
Yes	785	98.1
No	15	1.9
Symptoms of drug use*		
Kidney failure	540	67.5
Brain damage	520	65.0
Dry mouth	287	35.9
Visual hallucinations	169	21.1
I don’t know	90	11.3
Auditory hallucinations	125	15.6
Constipation	39	4.9
Diarrhoea	24	3.0
Mental disorders	12	1.5
No symptom or complication	3	0.4
Short-term complications of drug use*		
Dependence	246	30.8
Anxiety and depression	188	23.5
Sleep disorder	175	21.9
I don’t know	167	20.9
Aggressiveness	131	16.4
Personality disorder	99	12.4
Forgetfulness	98	12.3
Euphoria	94	11.8
Raised self-confidence	70	8.8
Improved memory	52	6.5
Pessimism	28	3.5
Long-term complications of drug use*		
I don’t know	311	38.9
Dependence	280	35.0
Anxiety and depression	134	16.8
Personality disorder	125	15.6
Aggressiveness	110	13.8
Sleep disorder	86	10.8
Forgetfulness	78	9.8
Raised self-confidence	46	5.8
Pessimism	37	4.6
Improved memory	34	4.3
Euphoria	29	3.6
Mental disorders	4	0.5

* , Multiple responses allowed.

The study found notable differences in addictive substance use based on age, sex and religion. A total of 5.4% of participants aged 12–14, 16.1% of those aged 15–17 and 33.3% of those aged 18+ had used addictive substances. A statistically significant association was found between age and substance use (*p* < 0.001). A total of 20.0% of male participants had used addictive substances, compared to 7.1% of female participants. A statistically significant association was observed between sex and substance use (*p* < 0.001). A total of 10.5% of Christian participants, 21.1% of Muslim participants and 33.3% of participants practising African traditional religion had used addictive substances. A statistically significant association was observed between religion and substance use (*p* < 0.001) (Table 4).

**TABLE 4:** Association between socio-demographic characteristics and addictive substance use among participants.

Variable	Addictive substance use				*x* ^2^	*P*
	Yes (*n* = 111)		No (*n* = 689)			
	Frequency	%	Frequency	%		
**Age (years)**						
12–14	14	5.4	245	94.6	35.641	0.000 (< 0.001)*
15–17	78	16.1	406	83.9	-	-
≥ 18	19	33.3	38	66.7	-	-
**Class**						
SS1	57	14.3	343	85.7	1.060	0.589
SS2	32	12.3	229	87.7	-	-
SS3	22	15.8	117	84.2	-	-
**Sex**						
Male	84	20.0	336	80.0	27.759	0.000 (< 0.001)*
Female	27	7.1	353	92.8	-	-
**Religion**						
Christianity	59	10.5	501	89.5	18.857	0.000 (< 0.001)*
Islam	48	21.1	180	78.9	-	-
Traditional	4	33.3	8	66.7	-	-

SS, Senior Secondary.

* , Statistically significant.

The study revealed that participants who used addictive substances had a higher mean family size (6.6 ± 2.5) compared to those who did not use substances (5.4 ± 1.4), with this difference being statistically significant (*p* < 0.001). In addition, over a third (38.9%) of participants whose mothers had no formal education used addictive substances, while less than a tenth (14.1%) of participants whose mothers had attained tertiary education did so. A statistically significant association was found between the mother’s education level and the participant’s substance use history (*p* < 0.001) (Table 5).

**TABLE 5:** Association between family details and addictive substance use among participants.

Variable	Addictive substance use								*x*^2^/*t*	*P*
	Yes (*n* = 111)				No (*n* = 689)					
	Frequency	%	Mean	s.d.	Frequency	%	Mean	s.d.		
**Family size**										
Mean ± s.d.	-	-	6.6	2.5	-	-	5.4	1.4	122.200	0.000 (< 0.001)*
**Father’s education level**										
No formal education	4	11.4	-	-	31	88.6	-	-	2.518	0.641
Primary	8	11.6	-	-	61	88.4	-	-	-	-
Secondary	36	12.2	-	-	260	87.8	-	-	-	-
Tertiary	34	15.2	-	-	190	84.8	-	-	-	-
I don’t know	29	16.5	-	-	147	83.5	-	-	-	-
**Mother’s education level**										
No formal education	14	38.9	-	-	22	61.1	-	-	125.034	0.000 (< 0.001)*
Primary	2	10.0	-	-	18	90.0	-	-	-	-
Secondary	33	9.6	-	-	311	90.4	-	-	-	-
Tertiary	19	9.1	-	-	189	90.9	-	-	-	-
I don’t know	27	14.1	-	-	165	85.9	-	-	-	-

s.d., standard deviation.

* , Statistically significant.

## Discussion

The results of the study showed that awareness of substance use was high among participants, with 98.1% reporting awareness. The study also found a strong level of knowledge about the effects of substance use. Most participants were able to identify the effects (88.7%) of substance use, both short-term (79.1%) and long-term (61.1%), complications associated with substance use. The negative effects most commonly recognised by participants included kidney failure, brain damage, dry mouth, visual hallucinations and auditory hallucinations. These findings align with similar studies conducted in Nigeria and other countries, which also report high levels of awareness regarding substance use.^14,27,28,29^

The prevalence of substance use among the participants in this study was 6.9% (current users only). About 13.9% of the participants had a history of substance use (lifetime prevalence). The prevalence of substance use in this study was lower than similar studies carried out in secondary schools in Jos, Plateau State and Oshogbo, Osun State, Nigeria, which revealed a substance abuse prevalence of 22.1% for a private school in Jos, 15.3% for a public school in Jos and 20.3% for Oshogbo secondary schools.^14,30^ Another study revealed a high prevalence of 47.9% with a lifetime prevalence of 65.4%.^31^

The lower prevalence of addictive substance use identified among participants in this study may be because of the continuous efforts of the Lagos State Government, Nigeria, in creating awareness about the dangers of substance use and training of teachers and school administrators on substance abuse prevention among secondary school students.^32^ Most of the schools visited during this study had an active guidance and counselling department, which holds regular seminars and interactive sessions with the students on the dangers of substance abuse.^32^ Recently, the Lagos State Ministry of Health, in conjunction with the MTN Foundation and a consortium of other stakeholders, unveiled an anti-drug abuse awareness project known as the Anti-Substance Abuse Awareness Programme (ASAP) to raise awareness on the menace of substance abuse and addiction in the state, especially among the youths and adolescents.^33^ Factors seen to be associated with the use of addictive substances in this study were age of the students (*p* = 0.000), gender (*p* = 0.000), religion (*p* = 0.000), the size of the family (*p* = 0.000) and the highest educational level of the mother (*p* = 0.000). Increasing age, male gender and increasing size of the family were seen to be positively associated with substance use, while the Christian religion and higher educational level of the mother were seen to be negatively associated with substance use.

The most common substances currently being used by the participants included tramadol, marijuana and shisha with a prevalence of 3.9%, 2.5% and 2%, respectively. Alcohol was the most frequently used substance, with over a fifth of the participants (22.6%) having ever drunk more than a sip of alcohol at one time or the other. Over a twentieth of the alcohol users (5.5%) drank alcohol for at least 20 days in the last 30 days before the study. A similar study in Southwest Nigeria also revealed tramadol as the most commonly abused substance apart from alcohol.^14^ Another study in the United States showed that in the 30 days prior to the survey, 41.2% of 12th graders had consumed alcohol and 19.2% of 12th graders had smoked tobacco cigarettes.^27^

The Youth Risk Behavior study conducted in 2015 in the United States also reported a prevalence of alcohol intake as high as 32.8%, which constituted respondents who had used alcohol on or before 30 days prior to the time of the study.^34^ Alcohol usage has been a long-standing and progressively rising concern among adolescents all over Africa. Another rising trend of substance abuse can be seen in the use of the prescription medication tramadol.^35^ Among the users of substance abuse in this study, tramadol was the most used (56.4%), then marijuana (36.4%) and shisha (29.1%). Other studies carried out in Nigeria also revealed tramadol as the most abused substance among secondary school students.^14,24,34^

### Strengths

The large sample size of 800 participants, the highly structured multistage sampling method and the use of previously validated WHO questionnaires are considered as strengths of this study.

### Limitations

This study was conducted among public secondary school students in Lagos State, a cosmopolitan and highly commercial state. The findings might not be exactly applicable to students in private schools and other states in Nigeria, thus limiting the generalisability of the study.

## Conclusion

The study shows a low prevalence of current substance use among the participants, although the lifetime prevalence is significantly higher. Apart from alcohol, tramadol was the most commonly used substance, followed by marijuana. This reiterates the need for stricter government policies regarding the accessibility of alcohol and tramadol to adolescents. This study revealed a high awareness of substance use and good knowledge of the effects of substance use among the participants. However, the knowledge of the long-term effects of substance use among the participants was lower than the knowledge of the short-term effects of substance use. The study reiterates the need for continuous awareness of the effects of substance abuse among adolescents, especially as it relates to long-term complications of substance use.

### Recommendations

Addressing the menace of substance use among youths and adolescents must be a holistic approach with government, schools, parents, non-governmental organisations (NGOs), religious bodies, communities and the entire society contributing their respective quota in creating an environment where substance abuse among youths and adolescents becomes totally eradicated or reduced to the minimum possible. Particularly, there is a need for further research and increased advocacy on the long-term complications of substance use among youths and adolescents. It is recommended that this study be replicated in different settings across Nigeria, such as rural areas and private schools, to explore variations in substance use patterns. Additionally, incorporating a qualitative component in future research would help identify the underlying reasons for substance use, offering deeper insights into the factors influencing students’ substance-use behaviour and informing more effective interventions.
